# Variation in Telehealth Use for Patients With Incident Atrial Fibrillation Across the Veterans Health Administration: Retrospective Cohort Study

**DOI:** 10.2196/76177

**Published:** 2025-10-28

**Authors:** Rebecca Lauren Tisdale, Neil M Kalwani, Harrison Koos, Jun Fan, Natasha Din, Alexander C Perino, David C Chan, Alexander Tarlochan Sandhu, Paul A Heidenreich

**Affiliations:** 1 VA Palo Alto Health Care System Menlo Park, CA United States; 2 Stanford University School of Medicine Stanford, CA United States; 3 University of California, Berkeley Berkeley, CA United States

**Keywords:** atrial fibrillation, telemedicine, remote consultation, videoconferencing, veterans, Veterans Health Administration, primary health care, cardiology, rural health services, health services accessibility

## Abstract

**Background:**

Telehealth is a potential tool to alleviate geographic clinician shortages, but there are limited data regarding current telehealth use for common cardiology conditions, including atrial fibrillation (AF).

**Objective:**

We aimed to evaluate variation in telehealth use in primary care and cardiology clinics for patients with incident AF in the Veterans Health Administration.

**Methods:**

We included patients diagnosed with AF in the outpatient setting between January 2022 and September 2023. We assessed the association between any video visit and any telehealth use (including phone) for primary care or cardiology visits within 90 days of an AF diagnosis, adjusting for selected patient- and facility-level characteristics using Bayesian logistic regression with facility-level random intercepts. We evaluated facility variation in video visit and telehealth use with the median odds ratio (MOR).

**Results:**

Our cohort included 36,929 patients with 80,596 visits across 125 facilities. Of the 63,835 primary care visits, 2088 (3.27%) were delivered via video and 13,403 (21%) via telehealth; of the 16,761 cardiology visits, 323 (1.93%) were delivered via video and 3288 (19.62%) via telehealth. On average, the mean age of the patients was 73.6 (SD 10.9) years; 2.91% (1075/36,929) were female; 77.71% (28,698/36,929) were White. In adjusted analyses, older age was associated with lower use of video visits for both primary and cardiology care and lower use of any telehealth for cardiology care (eg, adjusted odds ratio [AOR] 0.61, 95% credible interval [CrI] 0.42-0.85 for the use of video cardiology care for patients aged above 77 years). Living more than 65 km from the care site was associated with increased use of both video and any telehealth for primary and cardiology care (eg, AOR 1.91, 95% CrI 1.21-3.00 for video cardiology care); however, living in a rural location was associated with lower odds of using video or any telehealth for primary care (video: AOR 0.73, 95% CrI 0.64-0.84; telehealth: AOR 0.89, 95% CrI 0.83-0.96). There was marked variability across facilities in the use of video care (range 0%-17.4% of visits for cardiology care; 0%-12.5% for primary care) and telehealth (range 0%-82.6% for cardiology care; 3.8%-61.6% for primary care). The facility-level adjusted MOR for video care use was 1.97 (95% CrI 1.77-2.24) for primary care and 4.95 (95% CrI 3.39-7.98) for cardiology care. Similarly, the adjusted MOR for any telehealth use was 1.79 for primary care (95% CrI 1.65-1.96) and 2.61 for cardiology care (95% CrI 2.25-3.13).

**Conclusions:**

Following an incident AF diagnosis, telehealth may increase access to primary and cardiology care for veterans living at a distance, but its use remains lower for older patients and those in rural areas. There was substantial variation in telehealth use across facilities, which was not explained by differences in patient and facility characteristics. Standardizing telehealth use across Veterans Health Administration facilities may improve access to AF care.

## Introduction

### Background

The Veterans Health Administration (VHA) has promoted telehealth to broaden access to primary and specialty care and alleviate geographic clinician shortages. These efforts preceded the COVID-19 pandemic and enabled the rapid expansion of telehealth during the pandemic to maintain access to care [1]. Telehealth use remains more prevalent, but there are limited data on current patterns of virtual care in the VHA, especially for the management of specific cardiovascular conditions [2].

Atrial fibrillation (AF) is the most common cardiac rhythm disorder in the United States, affecting more than 6 million people, and it is associated with significant morbidity and cost [3,4]. Previous studies suggest that telehealth use for arrhythmia management may be higher than for other cardiovascular conditions [5,6], perhaps due to the availability of remote diagnostics such as ambulatory electrocardiogram monitoring. Therefore, telehealth may be an effective tool to increase access to AF care in the VHA, which serves patients across diverse geographic regions. Guidelines for AF management emphasize timely risk stratification and initiation of anticoagulation, rhythm or rate control, and risk factor management soon after AF diagnosis [7], and telehealth may increase access to AF care in this period.

Previous studies of telehealth use in the VHA early in the COVID-19 pandemic highlighted barriers to accessing video-based care for older patients and those living in rural areas [1,8,9]. Outside the VHA, cardiovascular cohorts similarly showed lower video visit use among older adults and those living in lower-income areas during the COVID-19 pandemic [10,11]. Evaluations of variation in telehealth use within cardiology care during the pandemic suggested that it was driven by patient factors and differences in physician preference, with 1 VHA analysis showing a lower contribution from facility-level differences [12,13]. However, another study of VHA primary care visits demonstrated substantial variation in video visit adoption at the facility level, with lower use at facilities serving more rural veterans [14]. Additional evidence is needed to assess current patterns of telehealth use, especially for prevalent conditions such as AF, which may be amenable to virtual management.

### Objectives

Characterizing the current state of telehealth use will be critical to identify opportunities for improvement within large integrated health systems such as the VHA. This is particularly relevant in the post–COVID-19 pandemic period, as telehealth is now an option rather than a requirement. Therefore, it is more urgent to define when telehealth is an acceptable, or even preferable, alternative to in-person care.

To address this gap, we characterized variation in telehealth use across patients and facilities within the VHA following new diagnoses of AF. We first identified patient- and facility-level factors associated with the likelihood of using telehealth and video care specifically and then quantified facility-level variation in telehealth use.

## Methods

### Data

We extracted data on a retrospective, nationwide cohort of VHA patients aged 18 years and older who were first diagnosed with AF in the outpatient setting between October 2018 and September 2023. We limited the sample to patients with newly diagnosed AF, as most patient education, diagnostic testing, and active clinical management for this condition occur at the time of diagnosis (eg, initiation of anticoagulation).

All data were derived from VHA’s corporate data warehouse. AF was defined as a primary or secondary diagnosis with AF-specific *International Classification of Diseases, 10th Revision* codes (Table S1 in Multimedia Appendix 1) recorded at an evaluation and management encounter in the outpatient setting. To improve specificity, following conventions of previous literature [15], a second AF diagnosis needed to be coded within 1 year after the index diagnosis for inclusion in the cohort. This second diagnosis could be recorded at an evaluation and management encounter or associated with a procedure code. To limit our sample to new diagnoses, in keeping with previous literature focused on incident AF [16], we excluded veterans with previous AF diagnoses coded in the 2 years before the index diagnosis.

We captured visits for cohort patients taking place in VHA-based primary care or cardiology clinics within the 90 days following an AF diagnosis. These visits were identified using primary care–specific and cardiology-specific VHA stop codes, which are 3-digit codes defined by the managerial accounting system of VHA that characterize all VHA outpatient encounters. We used current procedural terminology codes (Table S1 in Multimedia Appendix 1) to include only encounters representing office-type visits rather than other forms of patient contact (eg, phone calls to communicate results of laboratory testing). In addition, we classified visits as taking place via phone and video or in person using additional VHA stop codes for telehealth visits (Table S1 in Multimedia Appendix 1). Visits with conflicting labels at the same time were dropped (n=10).

In the VHA, a “facility” consists of a medical center and associated community-based clinics. Facilities with fewer than 20 visits for this period in the dataset and those located in non-US regions were excluded from analysis, as were patients receiving specialty care from multiple facilities, to allow patients to be attributed to a single facility for analytic models.

We captured selected veteran characteristics according to conventions of previous VHA telehealth literature focused on cardiovascular disease [8]. Patients with missing data were assigned to an unknown category for the variable in question. Age at the time of AF diagnosis was grouped into approximate tertiles for the study population, with resulting categories defined as younger than 70 years, aged 70 to 77 years, or older than 77 years. We captured race and ethnicity according to the most frequently recorded race or ethnicity identification in the electronic health record, with categories for race consisting of American Indian or Alaska Native, Asian, Black or African American, Native Hawaiian or other Pacific Islander, unknown, or White, and categories for ethnicity consisting of Hispanic or Latino, not Hispanic or Latino, or unknown. Rurality was defined according to US Census Bureau criteria [17] into categories of highly rural, rural, urban, or unknown; patients with rurality categorized as “I” for insular island were excluded from the analysis and captured in the year closest to the index visit. We captured driving distance to closest secondary care for patients who had cardiology visits and driving distance to closest primary care for patients who had primary care visits, dividing these distances into categories of short (<16 km [<10 miles]), medium (16-65 km [10-40 miles]), long (>65 km [>40 miles]), or missing; driving distance was derived from the year of data closest to the index visit. We included data regarding cardiovascular disease–related comorbidities of the patients, including hypertension, heart failure, stroke or transient ischemic attack, diabetes, myocardial infarction, coronary artery disease, peripheral artery disease, and chronic kidney disease in the 2 years before the index date (Table S1 in Multimedia Appendix 1). In addition, we captured the congestive heart failure, hypertension, aged 75 years or more, diabetes mellitus, stroke, vascular disease, aged 65 to 74 years, and sex category (CHA_2_DS_2_-VASc) score of patients, a measure of AF-related stroke risk intended to guide clinicians’ decision-making with respect to medical management of AF (ie, anticoagulation prescribing) [18]. Finally, we recorded the number of visits the patient had in primary care or cardiology clinic within 90 days of the incident AF diagnosis, categorized as 1, 2, 3, or 4 or more visits. We also captured facility-level characteristics, including facility complexity level [19,20], teaching hospital status, population size served, number of beds, and geographic region.

### Analysis

We plotted the percentage of primary care and cardiology visits within 90 days of AF diagnosis that were delivered via telehealth, consisting of video and phone, over time, including trends before and after the COVID-19 pandemic.

The primary analysis was limited to patients with AF diagnoses from January 2022 to September 2023 and visits within 90 days to capture a period at the end of and after the COVID-19 pandemic, which may be more generalizable to contemporary telehealth use.

We described sociodemographic and clinical characteristics of patients who had primary care and cardiology visits. As these are not independent groups (most patients who had cardiology visits also had primary care visits), we did not test the statistical significance of differences between these groups.

We estimated the association between patient and facility-level characteristics and receipt of video care or any telehealth (video or phone) using Bayesian mixed-effects logistic regression models with facility-level random intercepts. Patient- and facility-level characteristics were selected based on previous literature suggesting relationships between these characteristics and the use of telehealth [1,8]. Patient-level characteristics included age, sex, race and ethnicity, rurality, and driving distance to care. Furthermore, we adjusted for the selected comorbidities noted earlier and the patient’s number of primary care and cardiology visits within 90 days of the incident AF diagnosis. Facility-level characteristics included VHA facility complexity level, teaching status, patient population, number of hospital beds, and geographic region as noted previously. We hypothesized that facilities that were of higher complexity, academically affiliated, and larger would be more likely to use telehealth visits. Adjusted odds ratios (AORs) were reported with 95% credible intervals (CrIs).

To assess facility-level variation in video care and telehealth use, we compared the percentage of visits delivered through these modalities in primary care and cardiology clinics across VHA facilities. We also used the mixed effects models described earlier to calculate the median odds ratios (MORs) for receipt of video care and any telehealth after adjusting for patient- and facility-level characteristics. The MOR estimates the variation in the likelihood that 2 similar patients presenting to 2 similar facilities would receive video care or any telehealth [21]. For example, an MOR of 1.6 would indicate a typical 1.6-fold difference in the likelihood of video care or telehealth use between random sites. We used a 2-sided α of .05 to define statistical significance. Analyses were performed using R software (version 4.5.0; R Foundation for Statistical Computing).

### Ethical Considerations

This evaluation was conducted as a nonresearch quality improvement project supported by the US Department of Veterans Affairs Office of Connected Care, with the primary goal of assessing and improving VHA care; therefore, it was considered non–human participant research. It was thus exempt from human participant research ethics review and institutional review board approval. Patients were not compensated as data had been collected in the course of usual clinical operations and used for quality improvement, and informed consent was waived. All data analysis took place on a secure server to ensure the privacy and confidentiality of patients’ data.

## Results

### Overview

The overall cohort, including patients diagnosed with AF from October 2018 to September 2023, comprised 92,192 patients seen in primary care clinic with 176,572 visits within 90 days and 28,442 seen in cardiology clinic with 45,387 visits within 90 days. The analytic cohort of patients diagnosed with AF from January 2022 to September 2023 included 36,929 unique patients with 80,596 visits across 125 facilities (Table 1). There were 34,535 (93.52%) patients seen in primary care clinic within the 90 days following diagnosis, with 63,835 visits. There were 10,657 (28.86%) patients seen in cardiology clinic within the 90 days after diagnosis, with 16,761 total cardiology visits.

Among patients in the analytic cohort, 2.91% (1075/36,929) were female, 77.71% (28,698/36,929) were White, and 62.5% (23,090/36,929) resided in urban areas. Mean age was 73.6 (SD 10.9) years, and mean CHA_2_DS_2_-VASc score was 2.76 (SD 1.60). In total, 2088 (3.27%) of the 63,835 primary care visits and 323 (1.93%) of the 16,761 cardiology visits were delivered via video; 13,404 (21%) primary care visits and 3288 (19.62%) cardiology visits were delivered via telehealth. Facility-level characteristics are summarized in Table S2 in Multimedia Appendix 1.

Patients seen in cardiology clinic had qualitatively higher mean CHA_2_DS_2_-VASc scores (2.99 vs 2.74) and higher rates of all comorbidities captured compared to patients seen in primary care clinic. Patients with cardiology visits were more likely to be seen at facilities deemed to be of very high or high complexity, with 88.06% (9385/10,657) of patients with cardiology visits seen at these facilities compared to 79.32% (27,392/34,535) of patients with primary care visits.

**Figure 1:**
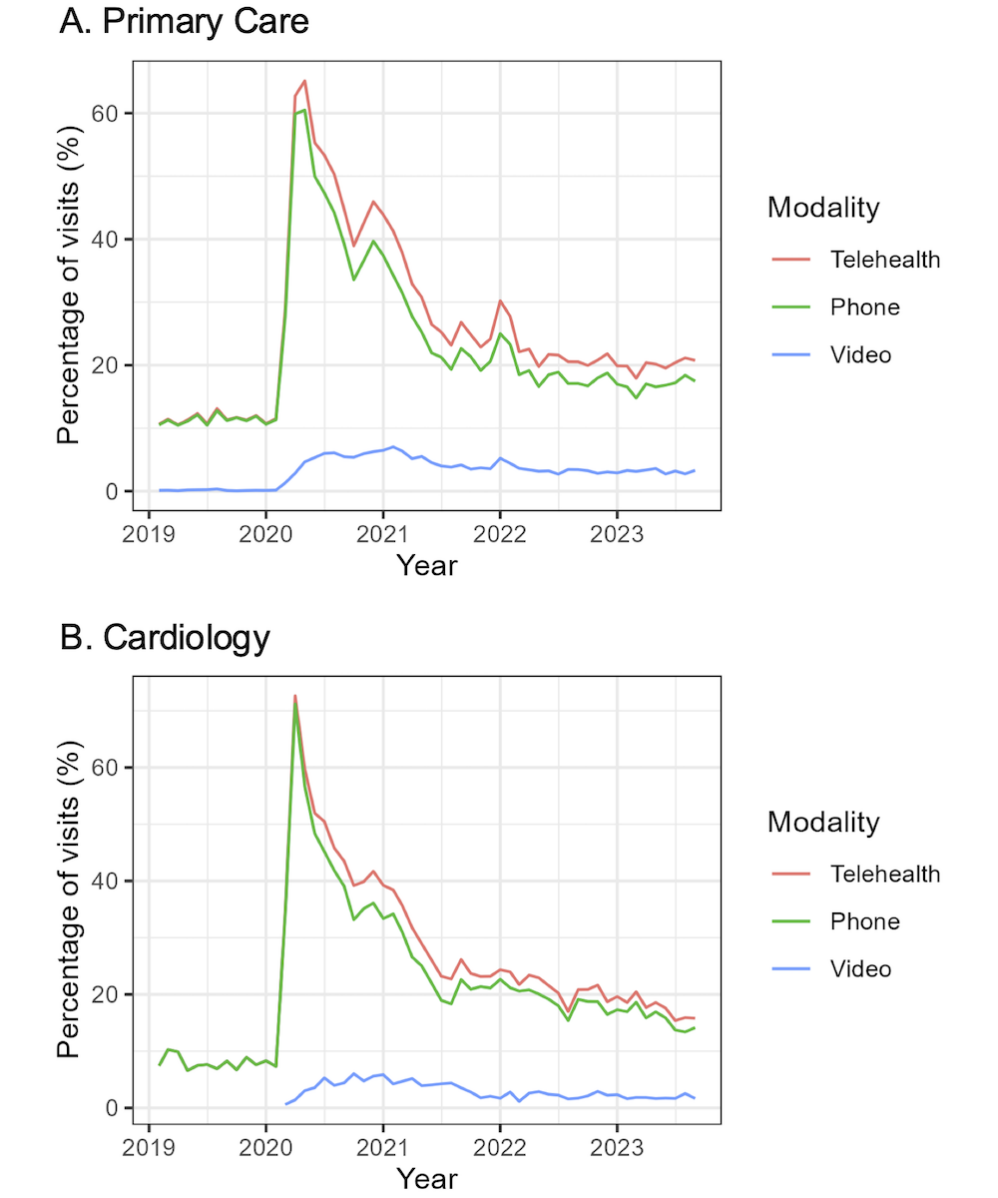
Percentage of visits offered via phone, video, or all telehealth modes within 90 days of new outpatient diagnoses of atrial fibrillation among veterans nationwide from October 2018 to September 2023: (A) primary care (n=176,572 visits) and (B) cardiology care (n=45,387 visits).

Figure 1 depicts the percentage of care offered via different modalities over time for the overall cohort. After initially peaking at the start of the COVID-19 pandemic and subsequently dropping, the relative proportions of care offered via phone and video versus in person remained reasonably stable for both primary and cardiology care, though perhaps with a slight downtrend for cardiology care after approximately mid-2022. Table S3 in Multimedia Appendix 1 provides the monthly visit counts.

**Table 1:** Patient-level characteristics for a nationwide cohort of veterans with new outpatient diagnoses of atrial fibrillation from January 2022 to September 2023^a^.

Field and dimensions			Overall (N=36,929)^b^		Cardiology care (n=10,657)^c^		Primary care (n=34,535)^d^
**Age (y)**							
	Mean (SD)	73.6 (10.9)		71.8 (10.6)		73.7 (10.9)	
	≤70, n (%)	11,512 (31.17)		3962 (37.18)		10,618 (30.75)	
	70-77, n (%)	13,002 (35.21)		3863 (36.25)		12,181 (35.27)	
	>77, n (%)	12,415 (33.62)		2832 (26.57)		11,736 (33.98)	
**Sex,** **n (%)**							
	Male	35,854 (97.09)		10,293 (96.58)		33,542 (97.12)	
	Female	1075 (2.91)		364 (3.42)		993 (2.88)	
**Race,** **n (%)**							
	American Indian or Alaska Native	312 (0.84)		93 (0.87)		296 (0.86)	
	Asian	177 (0.48)		53 (0.5)		168 (0.49)	
	Black or African American	4429 (11.99)		1623 (15.23)		4057 (11.75)	
	Native Hawaiian or other Pacific Islander	265 (0.72)		73 (0.68)		252 (0.73)	
	White	28,698 (77.71)		8081 (75.83)		26,883 (77.84)	
	Unknown	3048 (8.25)		734 (6.89)		2879 (8.34)	
**Ethnicity** **,** **n (%)**							
	Hispanic or Latino	1280 (3.47)		396 (3.72)		1197 (3.47)	
	Not Hispanic or Latino	33,352 (90.31)		9737 (91.37)		31,159 (90.22)	
	Unknown	2297 (6.22)		524 (4.92)		2179 (6.31)	
**Distance to clinic (km)** **,** **n (%)**							
	<16 km	—^e^		2430 (22.8)		14,723 (42.63)	
	16-65 km	—		5226 (49.04)		16,617 (48.12)	
	>65 km	—		2970 (27.87)		2788 (8.07)	
	Unknown	416 (1.13)		31 (0.29)		407 (1.18)	
**Rurality status** **,** **n (%)**							
	Urban	23,090 (62.53)		7118 (66.79)		21,504 (62.27)	
	Rural	11,826 (32.02)		3149 (29.55)		11,112 (32.18)	
	Highly rural	1601 (4.34)		373 (3.5)		1512 (4.4)	
	Unknown	412 (1.12)		17 (0.16)		407 (1.18)	
**Included visits, n (%)**							
	1	20,571 (55.7)		7052 (66.17)		18,978 (54.95)	
	2	9307 (25.2)		2323 (21.8)		8762 (25.37)	
	3	3771 (10.21)		738 (6.93)		3611 (10.46)	
	≥4	3280 (8.88)		544 (5.1)		3184 (9.22)	
CHA_2_DS_2_-VASc^f^ score, mean (SD)			2.76 (1.60)		2.99 (1.67)		2.74 (1.58)
**Preexisting conditions** **,** **n (%)**							
	Hypertension	26,003 (70.41)		8544 (80.17)		24,086 (69.74)	
	Heart failure	7522 (20.37)		3052 (28.64)		6729 (19.48)	
	Stroke or transient ischemic attack	2861 (7.75)		939 (8.81)		2622 (7.59)	
	Diabetes	13,553 (36.7)		4466 (41.91)		12,554 (36.35)	
	Myocardial infarction	1981 (5.36)		894 (8.39)		1741 (5.04)	
	Chronic kidney disease	16,391 (44.39)		5396 (50.63)		15,168 (43.92)	
	Coronary artery disease	11,972 (32.42)		4311 (40.45)		10,892 (31.54)	
	Peripheral arterial disease	4806 (13.01)		1672 (15.69)		4405 (12.76)	
**Facility complexity level** **,** **n (%)**							
	Very high	16,350 (44.27)		5169 (48.5)		15,220 (44.07)	
	High	13,112 (35.51)		4216 (39.56)		12,172 (35.2)	
	Medium	3859 (10.45)		831 (7.8)		3661 (10.6)	
	Low	3440 (9.32)		405 (3.8)		3321 (9.62)	
	Unknown	168 (0.45)		36 (0.34)		161 (0.47)	
**Facility teaching status** **,** **n (%)**							
	Major teaching hospital	15,095 (40.88)		4859 (45.59)		13,981 (40.48)	
	Minor teaching hospital	16,948 (45.89)		4862 (45.62)		15,884 (45.99)	
	Nonteaching hospital	4427 (11.99)		901 (8.45)		4221 (12.22)	
	No hospital data	459 (1.24)		35 (0.33)		449 (1.3)	
**Facility patient population,** **n (%)**							
	≤30,000	4725 (12.79)		901 (8.45)		4518 (13.08)	
	30,000-50,000	14,024 (37.98)		4085 (38.33)		13,085 (37.89)	
	>50,000	18,180 (49.23)		5671 (53.21)		16,932 (49.03)	
**Facility beds, n (%)**							
	0	459 (1.24)		35 (0.33)		449 (1.3)	
	1-100	6324 (17.12)		1479 (13.88)		6009 (17.4)	
	101-500	22,536 (61.03)		6663 (62.52)		21,006 (60.82)	
	>500	7610 (20.61)		2480 (23.27)		7071 (20.47)	
**Facility region** **,** **n (%)**							
	South	16,715 (45.26)		4991 (46.83)		15,578 (45.12)	
	West	6999 (18.95)		1931 (18.12)		6583 (19.06)	
	Northeast	5427 (14.7)		1531 (14.37)		5097 (14.76)	
	Midwest	7788 (21.09)		2204 (20.68)		7277 (21.07)	

^a^Individual patients receiving both primary care and cardiology visits during the study period were represented in both subcohorts; hence, the total number of patients is smaller than the sum of patients seen in primary care and cardiology clinic.

^b^The overall number of visits was 80,596.

^c^The number of cardiology visits was 16,761.

^d^The number of primary care visits was 63,835.

^e^The distance to clinic was given as distance to the closest primary care site for the primary care column and as distance to the closest secondary care site for the cardiology care column. Since these are different metrics, we did not aggregate them for the overall column.

^f^CHA_2_DS_2_-VASc: stroke risk score including congestive heart failure, hypertension, aged 75 years or more, diabetes mellitus, stroke, vascular disease, aged 65 to 74 years, and sex category.

### Video Care

**Figure 2:**
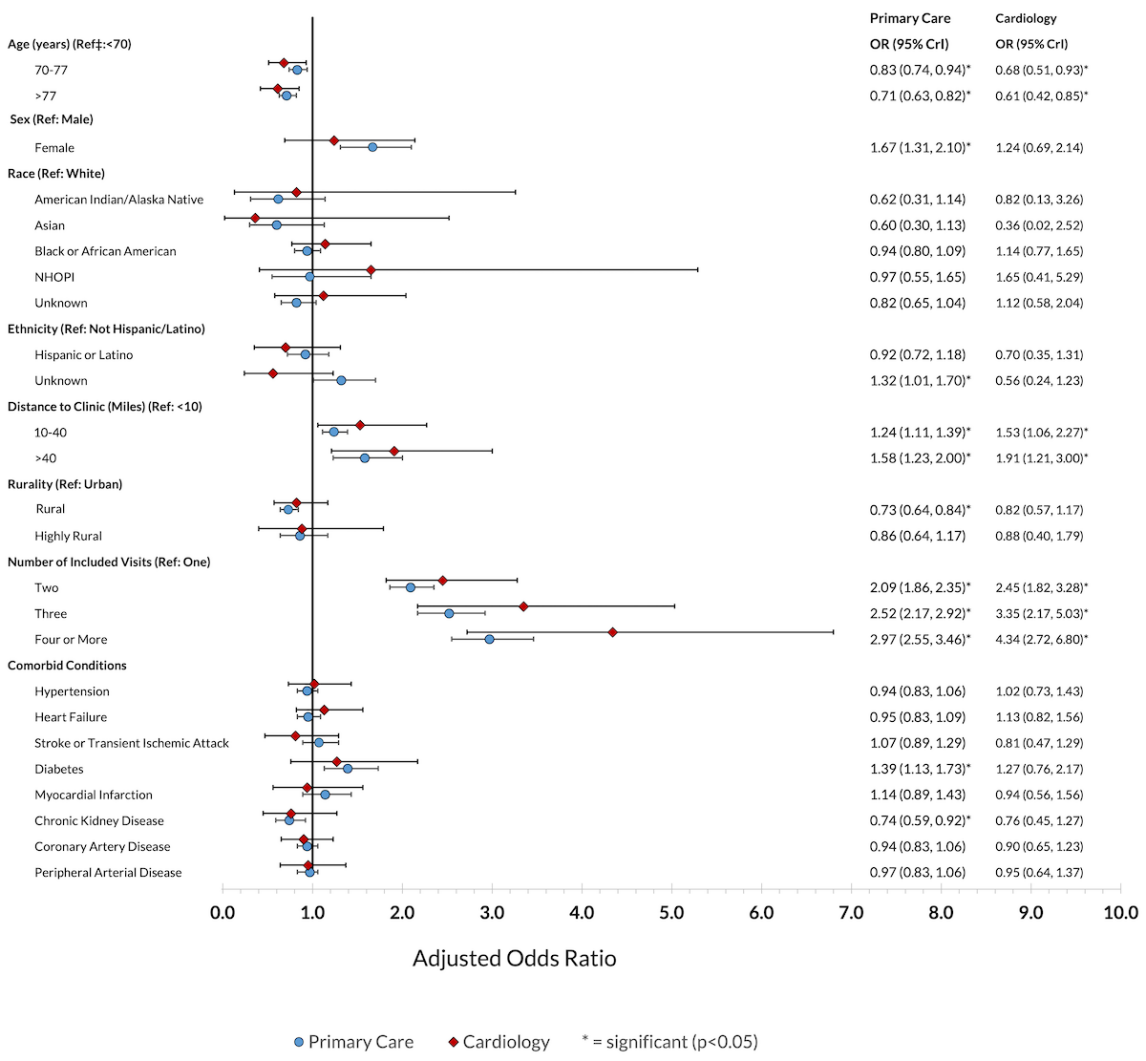
Adjusted odds ratios for the likelihood of any video care use within 90 days following new outpatient diagnoses of atrial fibrillation among veterans nationwide from January 2022 to September 2023 (34,535 patients with 63,835 primary care visits; 10,657 patients with 16,761 cardiology visits). CrI: credible interval; NHOPI: Native Hawaiian or other Pacific Islander; OR: odds ratio.

In the adjusted analysis, primary care video visit use was more likely among veterans who were younger (AOR for those aged >77 years 0.71, 95% CrI 0.63-0.82), were female (AOR 1.67, 95% CrI 1.31-2.10), had a diagnosis of diabetes (AOR 1.39, 95% CrI 1.13-1.73), or lived at a medium (16-65 km) or long (>65 km) distance from the site of care (AOR 1.24, 95% CrI 1.11-1.39 and AOR 1.58, 95% CrI 1.24-2.00, respectively) (Figure 2). Video care was less likely among patients with chronic kidney disease and those living in a rural setting. The odds of receiving at least 1 video visit increased as the patient’s total number of visits increased. No facility-level characteristics were associated with the likelihood of video care use (Table S4 in Multimedia Appendix 1).

For cardiology video-based care, similar patterns emerged regarding age, distance to care site, and number of visits (Figure 2). However, there was no difference by sex, chronic condition, or rurality. Patients receiving care at higher complexity facilities were less likely to use video visits (Table S4 in Multimedia Appendix 1).

**Figure 3:**
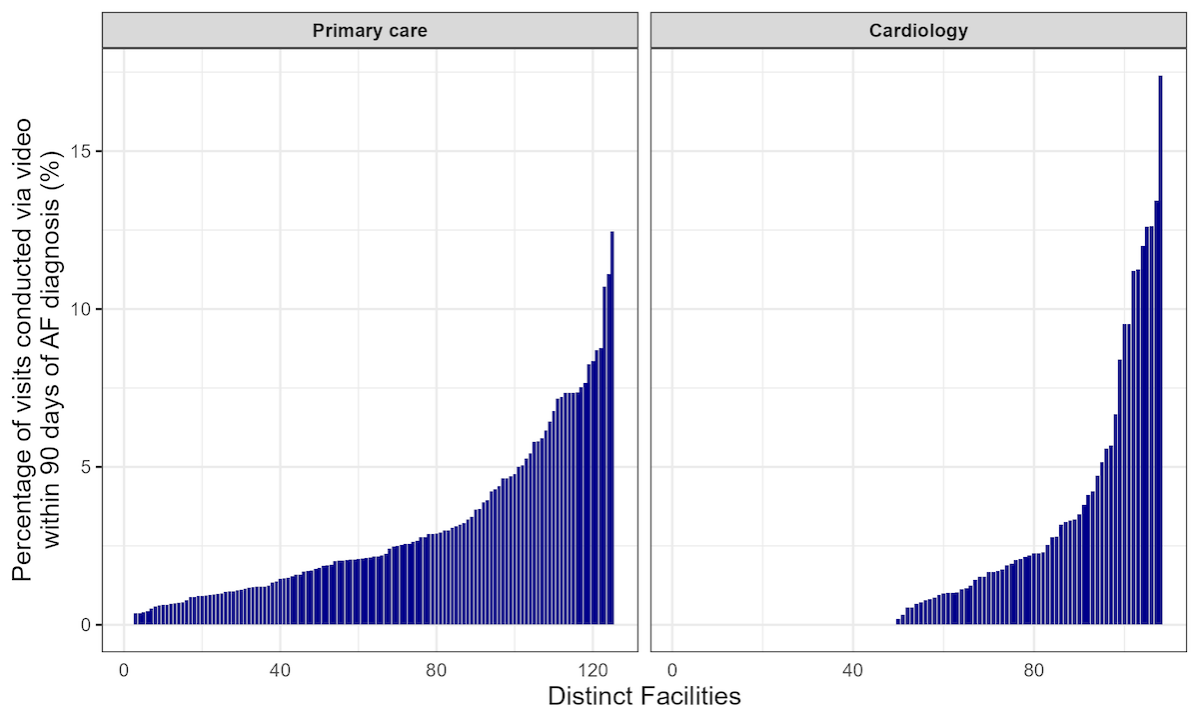
Variation in primary care and cardiology facility-level video care use for veterans nationwide with new outpatient diagnoses of atrial fibrillation (AF) from January 2022 to September 2023 (34,535 patients with 63,835 primary care visits; 10,657 patients with 16,761 cardiology visits). Each bar represents 1 facility.

There was significant variation in video care use across facilities for primary care visits (range 0%-12.5%; IQR 1.2%-4.2%) and cardiology visits (range 0%-17.4%; IQR 0%-2.3%). A substantial proportion (49/108, 45.4%) of cardiology practices provided no video care for patients with newly diagnosed AF (Figure 3). The adjusted MORs, which reflected the typical difference in the likelihood that similar patients at similar facilities received any telehealth, also suggested large variation in the use of video care for these patients in primary care (MOR 1.97, 95% CrI 1.77-2.24) and cardiology clinic (MOR 4.95, 95% CrI 3.39-7.98).

### All Telehealth

**Figure 4:**
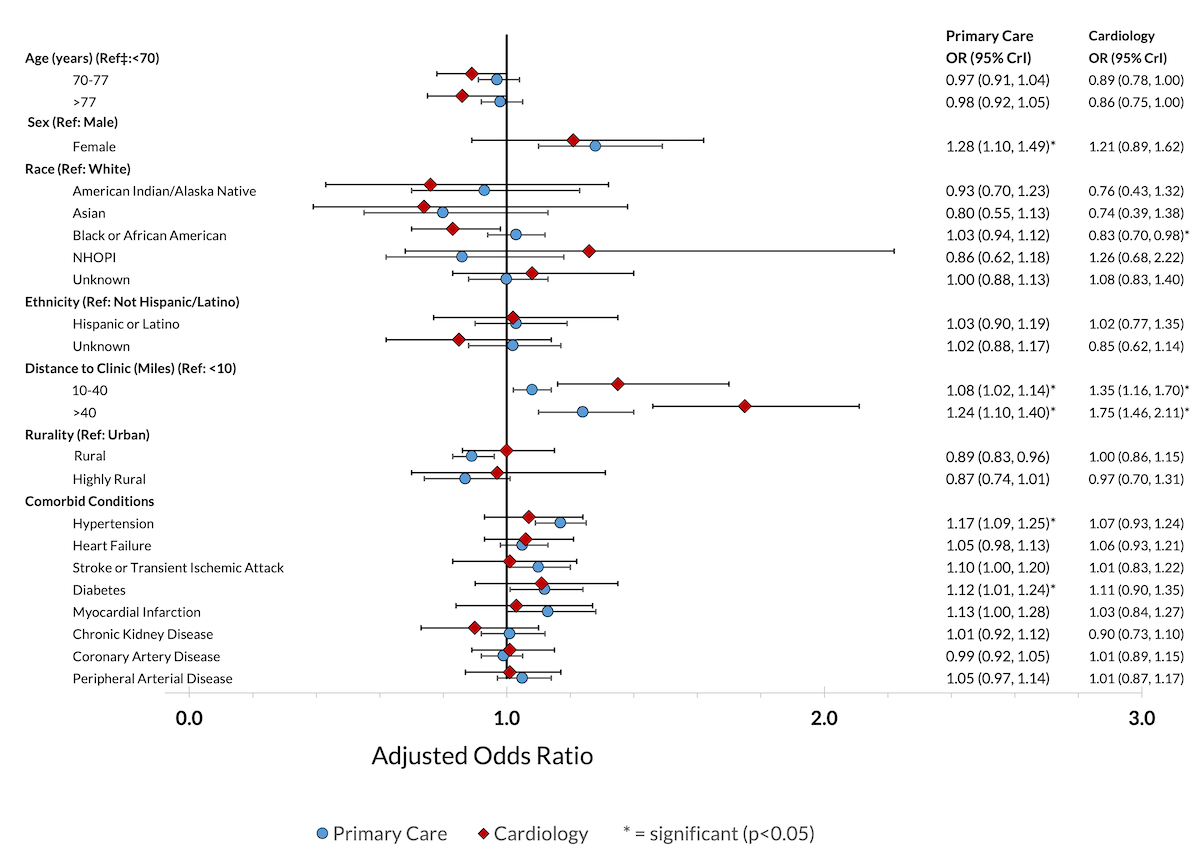
Adjusted odds ratios for the likelihood of any telehealth use within 90 days following new outpatient diagnoses of atrial fibrillation among veterans nationwide from January 2022 to September 2023 (34,535 patients with 63,835 primary care visits; 10,657 patients with 16,761 cardiology visits). CrI: credible interval; NHOPI: Native Hawaiian or other Pacific Islander; OR: odds ratio.

In the adjusted analysis, there was no association of telehealth use with age (Figure 4). Similar to the results for video care, female veterans were more likely to receive telehealth for primary care (AOR 1.28, 95% CrI 1.10-1.49) but not for cardiology care, and living at a moderate or long distance from care was associated with greater telehealth use for both primary care (AOR 1.08, 95% CrI 1.02-1.14 and AOR 1.24, 95% CrI 1.10-1.40, respectively) and cardiology care (AOR 1.35, 95% CrI 1.16-1.70 and AOR 1.75, 95% CrI 1.46-2.11, respectively), with a larger effect for cardiology care. Patients receiving a greater number of visits were more likely to receive any telehealth in both specialties (Table S4 in Multimedia Appendix 1). Having a diagnosis of hypertension or diabetes was associated with a greater likelihood of primary care telehealth use (Figure 4). Regarding facility-level characteristics, patients receiving care at higher-complexity facilities were less likely to use telehealth for cardiology care but not for primary care, and patients receiving care at facilities located in the west were more likely to use telehealth for both primary care and cardiology care (Table S4 in Multimedia Appendix 1).

**Figure 5:**
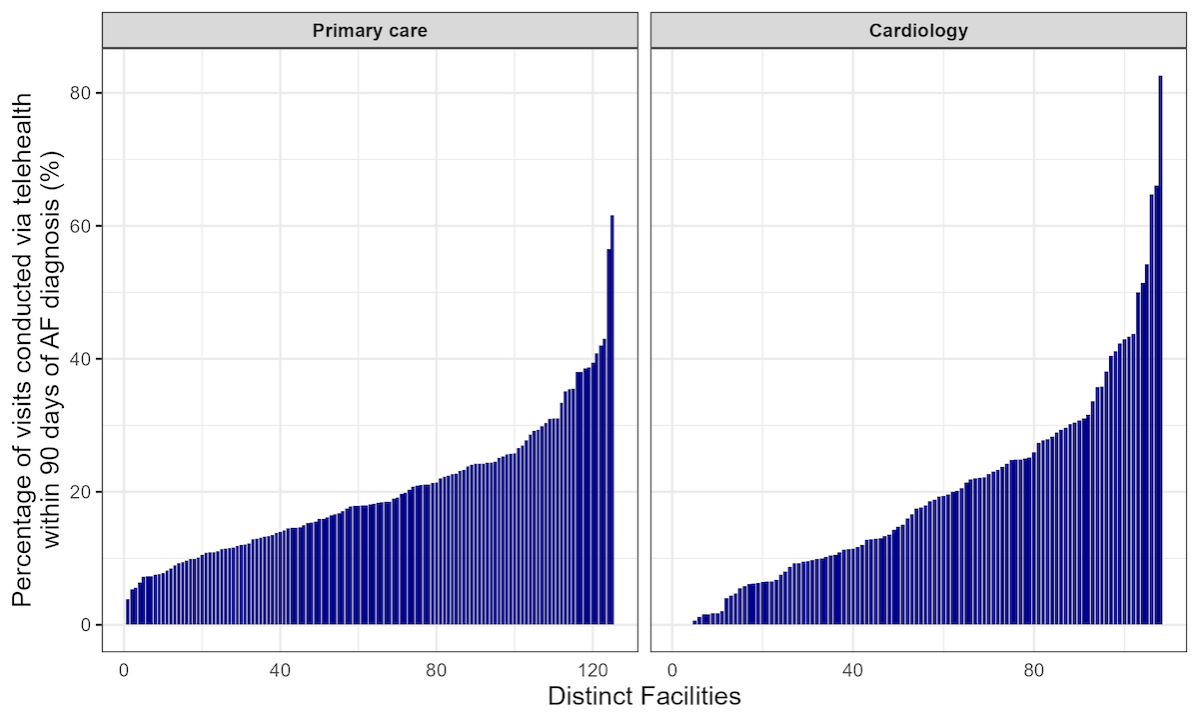
Variation in primary care and cardiology facility-level telehealth use for veterans nationwide with new outpatient diagnoses of atrial fibrillation (AF) from January 2022 to September 2023 (34,535 patients with 63,835 primary care visits; 10,657 patients with 16,761 cardiology visits). Each bar represents 1 facility.

There was substantial variation across facilities in overall telehealth use for patients with newly diagnosed AF with a wider range than for video care use. Telehealth use for primary care ranged from 3.8% to 61.6% of visits (IQR 12.2%-24.4%) and for cardiology care from 0% to 82.6% (IQR 9.3% to 27.5%; Figure 5). The adjusted MOR for any telehealth use was 1.78 (95% CrI 1.65-1.96) for primary care and 2.61 (95% CrI 2.25-3.13) for cardiology care.

## Discussion

### Principal Findings

In this investigation of care modalities for veterans within 90 days of an incident AF diagnosis, we found that about one-fifth of primary care and cardiology visits were provided through telehealth during this period at the end of and after the COVID-19 pandemic. Overall, video care use remained low, representing only 2% to 3% of visits, and was less likely among older patients and those living in rural areas receiving primary care. Video care and all telehealth use were more likely for those living at a distance from care. Importantly, there was significant variation in video and all telehealth use across VHA facilities even after adjusting for patient and facility-level characteristics.

In contrast to much of the existing literature on telehealth for patients with cardiovascular disease, this study focuses on a narrowly defined population of patients within 90 days of a new diagnosis of a specific cardiovascular condition. Despite this consistent clinical context, telehealth use across VHA facilities ranged from approximately 4% to more than 60% of the primary care visits and from 0% to more than 80% of the cardiology visits for this patient population. Notably, more than 45% of VHA cardiology practices did not use any video care for these patients, suggesting potential gaps in access to video care for patients newly diagnosed with AF. This variation was not explained by observable patient and facility factors, with a 5-fold difference in the likelihood that 2 similar patients presenting to 2 similar facilities with a new AF diagnosis would receive video-based cardiology care. This finding underscores the need for further investigation into facility-level policies, infrastructure, and clinician preferences that may influence telehealth adoption and the possible quality of care differences that accompany this substantial variation across facilities [22]. The persistence of these significant differences suggests that additional efforts are needed, particularly in rural areas, to fully leverage the potential of telehealth in expanding access to cardiovascular care.

### Comparison to Prior Work

Our findings align with previous research indicating that telehealth use in the VHA has stabilized at a higher level than before the COVID-19 pandemic, even years after its historic peak, reflecting both patient and clinician preference to continue integrating these modalities in addition to in-person care [2]. This mirrors trends seen outside of the VHA in the United States and globally, with an analysis of 20 countries showing telehealth use accounting for 19% of physician consultations in 2021 [23,24]. As shown in previous analyses outside of the VHA [11], we found that video care and all telehealth use were more likely for patients living at a distance from the site of care, highlighting the potential of these modalities to increase access for patients with geographic barriers to care, a critical goal for the VHA, which serves veterans across the entire nation. Of note, this distance effect was stronger for cardiology visits; this suggests that telehealth could be more effective for improving access to cardiology care, which is more geographically restricted than primary care.

However, inequities in telehealth access persist. Older veterans with incident AF were less likely to receive video visits in the postdiagnosis period, and rural veterans were less likely to receive primary care video visits—findings that align with previous analyses within and outside of the VHA [8,9,11,23,25]. These disparities are troubling, given that individuals aged 75 years and older have the highest prevalence of AF [3]—and therefore likely the highest need for care—and that rural veterans tend to face greater barriers to accessing care than their urban counterparts [26]. Video-based care offers specific advantages over audio-only visits, including the ability to conduct a visual physical examination, to assess a patient’s home environment and build a connection with patients through visual emotional cues (eg, facial expressions). Conversely, female veterans were more likely to use both video care and any telehealth. This is consistent with previous findings regarding female veterans’ telehealth use [1,8] and suggests that telehealth may specifically increase access for these patients who may face unique barriers to accessing in-person care, such as greater caregiving responsibilities [27].

Previous analyses of cardiology telehealth use during the COVID-19 pandemic have suggested that most variation in telehealth use is explained by patient and clinician factors, with 1 VHA analysis demonstrating less variation at the facility level [12,13]. In contrast, another study of VHA primary care video visit use early in the COVID-19 pandemic demonstrated significant variation across VHA facilities [14]. In this study, we found substantial variation in all telehealth and video visit use following AF diagnosis across facilities that was unexplained by patient- and facility-level characteristics. This degree of variation is notable as it occurred despite the VHA’s status as an integrated system with universal telehealth coverage and policies (such as cross-state practice) that decrease barriers to telehealth implementation [28]. This variation could be due to distinct clinician preferences for telehealth across sites or differences in local operational choices. Studies outside the United States have demonstrated that implementation of virtual care pathways for AF management supported by mobile health technology is feasible and may improve clinical outcomes [29,30]. Standardizing telehealth use across VHA facilities may thus represent an opportunity to improve both care access and quality for veterans with newly diagnosed AF.

### Future Directions

The VHA has actively expanded its telehealth capabilities to improve access to care, including through the use of telehealth hub-and-spoke networks designed to increase clinician supply in resource-limited areas [31]. These networks enable longitudinal clinical relationships even when veterans and clinicians are based at different facilities. For such initiatives to succeed, it is crucial to better understand the factors driving variation in telehealth use across facilities, particularly in the early management of AF—a common and costly condition requiring timely intervention, which may be well-suited to virtual management. Understanding why telehealth use is limited for patients with AF at some facilities will be essential in the quest to connect patients with adequate specialty care. Future research must also focus on assessing the quality and outcomes of telehealth care provided for cardiovascular conditions such as AF. In particular, exploring clinical outcomes such as appropriate anticoagulation prescription, stroke prevention, and acute care use will link access to meaningful care quality. The observed variation in telehealth adoption, which is unexplained by patient- and facility-level factors, offers a potential natural experiment to investigate the impact of telehealth on care delivery. Finally, targeted interventions may be needed to realize the potential of telehealth to enhance care delivery and ensure equitable telehealth access, particularly for older and rural veterans. These could include technology training or equipment access programs [32-34] and broadband access initiatives [35-37].

### Limitations

Our study has limitations. First, the data are restricted to VHA encounters; therefore, they do not account for care received outside the VHA system. It is possible that patients receiving care for existing diagnoses of AF outside of the VHA who then transferred their care to the VHA during the study period may have been misidentified as having incident AF. However, our large sample size of VHA-based care is a relative strength. Second, our VHA-based findings may not be generalizable to other contexts, particularly private sector or mixed-payer settings, and patient populations. In particular, women are underrepresented in the VHA compared to the general population, which limits insights into sex disparities outside the VHA system. Characterizing telehealth use in these settings and populations may require dedicated studies. Nonetheless, our results offer insights that may be relevant for other integrated health care systems. Third, while we included all primary care and cardiology visits in the immediate post-AF diagnosis period, AF may not have been the primary focus of these encounters. Our data do not allow for characterization of which visits were specifically focused on AF, although we hope to incorporate some indication of whether AF was managed at a given visit in future work. Fourth, as the analytic cohort spanned a period from the late COVID-19 pandemic to after the COVID-19 pandemic, there may have been pandemic effects that influenced the associations described. We minimize this risk to the extent possible by beginning our study period after telehealth proportions largely stabilized within the VHA system [2]. Fifth, data regarding certain known contributors to telehealth use, including socioeconomic status, broadband availability, digital literacy, and local COVID-19 burden, were not available for our study. As a result, our findings may be subject to residual confounding, although this is unlikely to explain the full range of variability across VHA facilities seen. Sixth, we may have misclassified visit modality in visits where video encounters were converted to phone visits or vice versa. Past work suggests that video-to-phone conversions are more common; therefore, our video estimates represent a likely upper bound [38]. Furthermore, such modality conversions would not affect our analyses incorporating all telehealth, as these combine phone and video care. Finally, our cross-sectional design limits causal inference, and we could not determine the etiologies of the observed differences in telehealth use. Future investigations should leverage natural experiments and mixed methods to better understand the drivers of variation in telehealth use.

### Conclusions

Telehealth accounted for a substantial proportion of care provided to this cohort of veterans with newly diagnosed AF, even in the late to post–COVID-19 pandemic period, suggesting relevance for care going forward. Telehealth use was more likely for those living at a distance, but video care rates remained low, and there was evidence of barriers in access for certain groups. Even after adjusting for patient- and facility-level factors, there was substantial variation in video care and all telehealth use between facilities. Standardizing telehealth use across the VHA may be an effective approach to enhancing access to care for AF—a highly prevalent condition with limited specialist availability.

## Appendix

Multimedia Appendix 1 Supplemental methods and results.

## Abbreviations

AF: atrial fibrillation
AOR: adjusted odds ratio
CHA2DS2-VASc score: congestive heart failure, hypertension, aged 75 years or more, diabetes mellitus, stroke, vascular disease, aged 65 to 74 years, and sex category score
CrI: credible interval
MOR: median odds ratio
VHA: Veterans Health Administration
