# Supplementary material for: Variation in Telehealth Use for Patients With Incident Atrial Fibrillation Across the Veterans Health Administration: Retrospective Cohort Study
Source: J Med Internet Res. 2025 Oct 28;27:e76177. doi: 10.2196/76177 (PMC12605270; doi:10.2196/76177)
Supplement: Multimedia Appendix 1 [file jmir_v27i1e76177_app1.docx]

Supplement to: Variation in Telehealth Use for Patients with Incident Atrial Fibrillation Across the Veterans Health Administration: Retrospective Cohort Study

Table S1: ICD10, CPT, and VA Stop Codes to Define Diagnoses and Visits

| **ICD-10 Codes** |  |
| --- | --- |
| **Atrial Fibrillation** | I480, I4811, I4819, I4820, I4821, I483, I484, I4891, I4892 |
| **Comorbidities** |  |
| **Coronary artery disease** | I25.XX; Z95.1; Z95.5; all MI codes |
| **Myocardial infarction** | I21.XX; 122.X; 123.X; 124.X |
| **Congestive Heart Failure** | I09.XX; I11.0; I13.X; I25.5; I42.XX; I43; I50.XX; I51.X |
| **Hypertension** | I10; I11.X; I12.X; I13.X; I15.X; I16.X |
| **Prior Bleed** | I60.XX; I61.XX; I62.XX; I69.0XX; I69.1XX; I69.2XX; S06.XX; I85.01; I85.11; K22.11; K22.6; K25.XX; K26.X; K27.X; K28.X; K29.X; K31.8X; K51.411; K55.21; K57.X; K62.5; K64.X; K66.1; K92.X; K94.01; K94.11; K94.21; K94.31; H05.23; H05.231; H05.232; H05.233; H05.239; H31.30; H31.301; H31.302; H31.303; H31.309; H31.31; H31.311; H31.312; H31.313; H31.319; H31.41; H31.422; H31.412; H31.413; H31.419; H35.6X; H35.73X; H43.1X; H44.8X; H47.02X; H60.32X; M25.0X; N30.01; N30.11; N30.21; N30.31; N30.41; N30.81; N30.91; N42.1; R31.0; R31.9; N99.520; N99.530; R04.0; R04.1; R04.2; R04.8; R04.89; R04.9; R58; T79.2; E07.89; E27.49; D62; D68.3 |
| **Anemia** | D50.X; D51.X; D52.X; D53.X; D55.X; D56.X; D57.X; D58.X; D59.X; D60.X; D61.X; D62; D63.X; D64.X |
| **Diabetes** | E08.XX; E09.XX; E10.XX ; E11.XX; E13.XX; Z96.41 |
| **Stroke/TIA** | I63.XX; I66.XX; I67.81; I67.82; I97.81; I97.810; I97.811; I97.82; I97.820; I97.821; G45.XX; I69.3XX |
| **Chronic Kidney Disease** | E08.2; E08.21; E08.22; E08.29; E09.2; E09.21; E09.22; E09.29; E10.2; E10.21; E10.22; E10.29 ;E11.2; E11.21; E11.22; E11.29; E13.2; E13.21; E13.22; E13.29; I12.X; I13.X; N18.X; N19 |
| **Kidney disease (varying chronicity)** | N17.X; N00.X; N01.X; N02.X; N03.X; N04.X; N05.X; N06.X; N07.X; N08; N11.X; N12; N4.X; N16; N19; N25.X |
| **Peripheral Arterial Disease** | I70.XX; I73.XX; K55.1X; K55.1; K55.9 |
| **CPT Codes** |  |
| **In person or Video** | Between 99202 and 99215 |
| **Phone** | 99441-99443 |
| **VA Stop Codes*** |  |
| **Primary Care** | 170, 301,318,319, 322, 323,324,326, 338,342, 348, 350, 352, 354 |
| **Cardiology** | 303 (in primary position unless 324 in primary position, in which case in secondary position) |
| **Phone** | 178 Telephone Hbpc, 324 Telephone/Medicine, 326 Telephone/Geriatrics, 338 Telephone Primary Care |
| **Video** | 179: Real Time Clinical Video Telehealth To Home - Provider Site, 648: Real Time Clinical Video Telehealth With Non-VAMC Location - Provider Site, 679: National Center Real Time Clinical Video Telehealth To Home- Provider Site |

*Virtual secure messaging stop codes were not included as we limited visits to those where AF management would most likely take place (i.e., evaluation and management office visits with a particular range of CPT codes).

Table S2. Facility characteristics

| **Field** | **Dimension** | **Count (%)** |
| --- | --- | --- |
|  | Total Stations | 125 (100.0%) |
| **Facility Complexity Level** | Very High | 38 (30.4%) |
|  | High | 40 (32.0%) |
|  | Medium | 19 (15.2%) |
|  | Low | 27 (21.6%) |
|  | Not applicable | 1 (0.8%) |
| **Facility Teaching Status** | Major Teaching Hospital | 41 (32.8%) |
|  | Minor Teaching Hospital | 54 (43.2%) |
|  | Non-Teaching Hospital | 24 (19.2%) |
|  | No Hospital/Hospital Data | 6 (4.8%) |
| **Facility Patient Population** | ≤30,000 | 34 (27.2%) |
|  | 30,000-50,000 | 49 (39.2%) |
|  | >50,000 | 42 (33.6%) |
| **Facility Beds** | 0 | 6 (4.8%) |
|  | 1-100 | 28 (22.4%) |
|  | 101-500 | 75 (60.0%) |
|  | >500 | 16 (12.8%) |
| **Facility Region** | S | 50 (40.0%) |
|  | W | 27 (21.6%) |
|  | NE | 22 (17.6%) |
|  | MW | 26 (20.8%) |

Table S3. Monthly primary care and cardiology visits among study cohort, 2/2019 – 9/2023

|  | **Visits (N)** | | |
| --- | --- | --- | --- |
| **Month** | **Cardiology** | **Primary Care** | **Overall** |
| **2019-02-01** | 850 | 3052 | 3902 |
| **2019-03-01** | 943 | 3404 | 4347 |
| **2019-04-01** | 961 | 3670 | 4631 |
| **2019-05-01** | 988 | 3583 | 4571 |
| **2019-06-01** | 869 | 3179 | 4048 |
| **2019-07-01** | 903 | 3369 | 4272 |
| **2019-08-01** | 869 | 3178 | 4047 |
| **2019-09-01** | 787 | 2873 | 3660 |
| **2019-10-01** | 878 | 3413 | 4291 |
| **2019-11-01** | 773 | 2853 | 3626 |
| **2019-12-01** | 750 | 2986 | 3736 |
| **2020-01-01** | 891 | 3390 | 4281 |
| **2020-02-01** | 793 | 3032 | 3825 |
| **2020-03-01** | 837 | 3037 | 3874 |
| **2020-04-01** | 705 | 2727 | 3432 |
| **2020-05-01** | 658 | 2298 | 2956 |
| **2020-06-01** | 645 | 2717 | 3362 |
| **2020-07-01** | 737 | 3200 | 3937 |
| **2020-08-01** | 858 | 3348 | 4206 |
| **2020-09-01** | 927 | 3784 | 4711 |
| **2020-10-01** | 962 | 3788 | 4750 |
| **2020-11-01** | 948 | 3324 | 4272 |
| **2020-12-01** | 964 | 3717 | 4681 |
| **2021-01-01** | 905 | 3623 | 4528 |
| **2021-02-01** | 898 | 3569 | 4467 |
| **2021-03-01** | 1112 | 4463 | 5575 |
| **2021-04-01** | 1083 | 4396 | 5479 |
| **2021-05-01** | 947 | 3894 | 4841 |
| **2021-06-01** | 1010 | 4021 | 5031 |
| **2021-07-01** | 823 | 3576 | 4399 |
| **2021-08-01** | 889 | 3681 | 4570 |
| **2021-09-01** | 849 | 3581 | 4430 |
| **2021-10-01** | 789 | 3537 | 4326 |
| **2021-11-01** | 842 | 3370 | 4212 |
| **2021-12-01** | 828 | 3398 | 4226 |
| **2022-01-01** | 821 | 3256 | 4077 |
| **2022-02-01** | 751 | 3304 | 4055 |
| **2022-03-01** | 952 | 3973 | 4925 |
| **2022-04-01** | 923 | 3617 | 4540 |
| **2022-05-01** | 872 | 3540 | 4412 |
| **2022-06-01** | 835 | 3382 | 4217 |
| **2022-07-01** | 711 | 3064 | 3775 |
| **2022-08-01** | 889 | 3386 | 4275 |
| **2022-09-01** | 758 | 3116 | 3874 |
| **2022-10-01** | 757 | 3046 | 3803 |
| **2022-11-01** | 790 | 3110 | 3900 |
| **2022-12-01** | 807 | 3108 | 3915 |
| **2023-01-01** | 861 | 3374 | 4235 |
| **2023-02-01** | 743 | 3189 | 3932 |
| **2023-03-01** | 977 | 3822 | 4799 |
| **2023-04-01** | 820 | 3063 | 3883 |
| **2023-05-01** | 969 | 3356 | 4325 |
| **2023-06-01** | 870 | 2711 | 3581 |
| **2023-07-01** | 721 | 2186 | 2907 |
| **2023-08-01** | 710 | 2217 | 2927 |
| **2023-09-01** | 601 | 1695 | 2296 |

Table S4. Adjusted odds ratios for the likelihood of any video care use or any telehealth use within 90 days following new outpatient diagnoses of atrial fibrillation among Veterans nationwide (N = 34,535 patients with primary care visits; 10,657 patients with cardiology visits; N = 63,835 primary care visits, 16,761 cardiology visits) from January 2022 to September 2023

|  |  | **Video Care** | | **Telehealth** | |
| --- | --- | --- | --- | --- | --- |
|  |  | **Primary Care** | **Cardiology** | **Primary Care** | **Cardiology** |
| **Field** | **Dimension** | **Odds Ratio (95% Credible Interval)** | | **Odds Ratio (95% Credible Interval)** | |
| **Age (years)** | <70 (reference) |  |  |  |  |
|  | 70-77 | 0.83 (0.74-0.94) | 0.68 (0.51-0.93) | 0.97 (0.91-1.04) | 0.89 (0.78-1.00) |
|  | >77 | 0.71 (0.63-0.82) | 0.61 (0.42-0.85) | 0.98 (0.92-1.05) | 0.86 (0.75-1.00) |
| **Sex** | Male (reference) |  |  |  |  |
|  | Female | 1.67 (1.31-2.10) | 1.24 (0.69-2.14) | 1.28 (1.10-1.49) | 1.21 (0.89-1.62) |
| **Race** | White (reference) |  |  |  |  |
|  | American Indian or  Alaska Native | 0.62 (0.31-1.14) | 0.82 (0.13-3.26) | 0.93 (0.70-1.23) | 0.76 (0.43-1.32) |
|  | Asian | 0.60 (0.30-1.13) | 0.36 (0.02-2.52) | 0.80 (0.55-1.13) | 0.74 (0.39-1.38) |
|  | Black or African  American | 0.94 (0.80-1.09) | 1.14 (0.77-1.65) | 1.03 (0.94-1.12) | 0.83 (0.70-0.98) |
|  | Native Hawaiian or  other Pacific Islander | 0.97 (0.55-1.65) | 1.65 (0.41-5.29) | 0.86 (0.62-1.18) | 1.26 (0.68-2.22) |
|  | Unknown | 0.82 (0.65-1.04) | 1.12 (0.58-2.04) | 1.00 (0.88-1.13) | 1.08 (0.83-1.40) |
| **Ethnicity** | Not Hispanic or Latino (reference) |  |  |  |  |
|  | Hispanic or Latino | 0.92 (0.72-1.18) | 0.70 (0.35-1.31) | 1.03 (0.90-1.19) | 1.02 (0.77-1.35) |
|  | Unknown | 1.32 (1.01-1.70) | 0.56 (0.24-1.23) | 1.02 (0.88-1.17) | 0.85 (0.62-1.14) |
| **Distance to Clinic (miles)*** | <10 (reference) |  |  |  |  |
|  | 10-40 | 1.24 (1.11-1.39) | 1.53 (1.06-2.27) | 1.08 (1.02-1.14) | 1.35 (1.16-1.70) |
|  | >40 | 1.58 (1.24-2.00) | 1.91 (1.21-3.00) | 1.24 (1.10-1.40) | 1.75 (1.46-2.11) |
| **Rurality Status*** | Urban (reference) |  |  |  |  |
|  | Rural | 0.73 (0.64-0.84) | 0.82 (0.57-1.17) | 0.89 (0.83-0.96) | 1.00 (0.86-1.15) |
|  | Highly Rural | 0.86 (0.64-1.17) | 0.88 (0.40-1.79) | 0.87 (0.74-1.01) | 0.97 (0.70-1.31) |
| **Number of Included Visits** | One (reference) |  |  |  |  |
|  | Two | 2.09 (1.86-2.35) | 2.45 (1.82-3.28) | 3.11 (2.93-3.31) | 3.79 (3.34-4.30) |
|  | Three | 2.52 (2.17-2.92) | 3.35 (2.17-5.03) | 5.02 (4.63-5.43) | 7.35 (6.14-8.83) |
|  | Four or More | 2.97 (2.55-3.46) | 4.34 (2.72-6.80) | 7.76 (7.12-8.48) | 12.1 (9.77-15.1**)** |
| **Comorbid Conditions** | Hypertension | 0.94 (0.83-1.06) | 1.02 (0.73-1.43) | 1.17 (1.09-1.25) | 1.07 (0.93-1.24) |
|  | Heart Failure | 0.95 (0.83-1.09) | 1.13 (0.82-1.56) | 1.05 (0.98-1.13) | 1.06 (0.93-1.21) |
|  | Stroke or Transient Ischemic Attack | 1.07 (0.89-1.29) | 0.81 (0.47-1.29) | 1.10 (1.00-1.20) | 1.01 (0.83-1.22) |
|  | Diabetes | 1.39 (1.13-1.73) | 1.27 (0.76-2.17) | 1.12 (1.01-1.24) | 1.11 (0.90-1.35) |
|  | Myocardial Infarction | 1.14 (0.89-1.43) | 0.94 (0.56-1.56) | 1.13 (1.00-1.28) | 1.03 (0.84-1.27) |
|  | Chronic Kidney Disease | 0.74 (0.59-0.92) | 0.76 (0.45-1.27) | 1.01 (0.92-1.12) | 0.90 (0.73-1.10) |
|  | Coronary Artery Disease | 0.94 (0.83-1.06) | 0.90 (0.65-1.23) | 0.99 (0.92-1.05) | 1.01 (0.89-1.15) |
|  | Peripheral Arterial Disease | 0.97 (0.83-1.06) | 0.95 (0.64-1.37) | 1.05 (0.97-1.14) | 1.01 (0.87-1.17) |
| **Facility Complexity Level** | Very High (reference) |  |  |  |  |
|  | Low | 0.82 (0.35-1.83) | 0.63 (0.04-7.99) | 0.90 (0.49-1.74) | 0.78 (0.21-2.95) |
|  | Medium | 0.55 (0.28-1.06) | 0.31 (0.05-1.85) | 0.74 (0.45-1.22) | 0.48 (0.21-1.19) |
|  | High | 0.68 (0.45-1.03) | 0.22 (0.07-0.66) | 1.07 (0.76-1.49) | 0.49 (0.28-0.86) |
|  | Not Available | 0.35 (0.05-2.35) | 0.00 (0.00-3.87) | 0.63 (0.17-2.31) | 0.07 (0.00-1.30) |
| **Facility Teaching Status*** | Major Teaching Hospital (reference) |  |  |  |  |
|  | Minor Teaching  Hospital | 0.98 (0.68-1.39) | 1.48 (0.54-4.11) | 1.02 (0.76-1.36) | 0.72 (0.43-1.20) |
|  | Non-Teaching Hospital | 0.84 (0.46-1.55) | 2.98 (0.57-16.9) | 1.02 (0.63-1.62) | 0.81 (0.34-2.03) |
| **Facility Patient Population** | ≤30,000 (reference) |  |  |  |  |
|  | 30,000-50,000 | 1.27 (0.76-2.06) | 1.12 (0.23-5.63) | 1.18 (0.79-1.72) | 1.11 (0.54-2.46) |
|  | >50,000 | 1.37 (0.74-2.54) | 1.85 (0.30-12.9) | 1.22 (0.75-1.99) | 0.79 (0.32-1.98) |
| **Facility Beds*** | 1-100 (reference) |  |  |  |  |
|  | 101-500 | 1.46 (0.97-2.21) | 1.19 (0.36-4.08) | 1.13 (0.82-1.56) | 0.94 (0.51-1.68) |
|  | >500 | 1.05 (0.56-1.94) | 0.55 (0.10-2.82) | 1.22 (0.76-1.97) | 0.89 (0.37-2.02) |
| **Facility Region** | South (reference) |  |  |  |  |
|  | West | 1.44 (0.98-2.14) | 1.46 (0.50-4.32) | 1.90 (1.42-2.59) | 2.31 (1.33-4.06) |
|  | Northeast | 1.58 (0.98-2.55) | 3.03 (0.74-14.0) | 1.03 (0.70-1.51) | 1.19 (0.59-2.43) |
|  | Midwest | 1.03 (0.69-1.54) | 1.53 (0.45-5.55) | 0.90 (0.65-1.23) | 0.71 (0.37-1.31) |

*Unknown distance not shown – video adjusted odds ratio [AOR] 0.62, 95% credible interval (CrI) 0.00-637,802 for primary care, AOR 0.01, 95% CrI 0.00-3.93 for cardiology; all telehealth AOR 0.38, 95% CrI 0.00-230,103 for primary care, AOR 0.29, 95% CrI 0.01-2.10 for cardiology.

Unknown rurality not shown – video AOR 0.71, 95% Cr I 0.00-1,204,772 for primary care, AOR 551.4, 95% Cr I 0.91-15,777,477 for cardiology; all telehealth AOR 0.36, 95% CrI 0.00-524,711 for primary care, AOR 11.3, 95% CrI 1.12-287.5 for cardiology.

No hospital data not shown – video AOR 1.14, 95% Cr I 0.00-1,313,265 for primary care, AOR 1.66, 95% Cr I 0.00-1,571,190 for cardiology; all telehealth AOR 1.36, 95% CrI 0.00-1,421,994 for primary care, AOR 1.18, 95% CrI 0.00-1,866,680 for cardiology.

Bed size 0 not shown – video AOR 1.07, 95% Cr I 0.00-1,153,255 for primary care, AOR 2.48, 95% CrI 0.00-1,588,608 for cardiology; all telehealth AOR 1.22, 95% CrI 0.00-1,375,451for primary care, AOR 1.03, 95% CrI 0.00-1,075,787 for cardiology.
